# Fixed Versus Flexible GnRH Antagonist Initiation in Women with Suboptimal Ovarian Response Undergoing IVF/ICSI: A Retrospective Cohort Study

**DOI:** 10.3390/jcm15218497

**Published:** 2026-11-01

**Authors:** Nina Medić, Damir Roje, Marina Šprem Goldštajn

**Affiliations:** 1 Department of Obstetrics and Gynecology, Clinical Hospital Sveti Duh, 10000 Zagreb, Croatia; 2 Polyclinic Hormona Split, 21000 Split, Croatia; rojedamir@gmail.com; 3 School of Medicine and Faculty of Health Sciences, University of Split, 21000 Split, Croatia; 4 Department of Obstetrics and Gynecology, Clinical Hospital Center Zagreb, 10000 Zagreb, Croatia; marina.goldstajn@gmail.com

**Keywords:** IVF, ICSI, GnRH antagonist, fixed protocol, flexible protocol, suboptimal ovarian response, live birth rate

## Abstract

**Background:** Suboptimal ovarian responders are a clinically important subgroup of patients treated with in vitro fertilization (IVF) or intracytoplasmic sperm injection (ICSI). Although their ovarian reserve is usually normal, fewer oocytes than expected are retrieved after controlled ovarian stimulation. Whether the timing of gonadotropin-releasing hormone (GnRH) antagonist initiation affects their reproductive outcomes is unknown. **Objective:** The objective of this study was to compare reproductive outcomes between fixed and flexible GnRH antagonist protocols in women with suboptimal ovarian response undergoing IVF/ICSI treatment. **Methods:** This retrospective single-center cohort study included 419 women treated with GnRH antagonist protocol between January 2019 and February 2020. After applying the exclusion criteria, 322 women undergoing IVF/ICSI with a GnRH antagonist protocol were eligible for analysis. Of these, 98 women with normal ovarian reserve and a suboptimal ovarian response (4–9 oocytes retrieved) were included, of whom 89 had a fresh embryo transfer (fixed protocol, n = 41; flexible protocol, n = 48). The primary outcome was live birth rate. Secondary outcomes included ovarian stimulation characteristics, embryo transfer strategy and pregnancy outcomes. Outcomes were analysed per embryo transfer and per oocyte retrieval, and factors associated with live birth were explored with Firth’s penalised logistic regression. **Results:** Baseline characteristics, including age, AMH, duration of infertility and number of previous IVF attempts, were comparable between groups. Antagonist initiation was later in the flexible group (median day 8 vs. 7), and more embryos were transferred in the fixed group (median 2 vs. 1). The numbers of retrieved and metaphase II oocytes did not differ. Live birth rate per transfer was higher with the fixed protocol (13/41, 31.7% vs. 6/48, 12.5%; *p* = 0.038), whereas positive β-hCG (43.9% vs. 27.1%; *p* = 0.12) and clinical pregnancy (41.5% vs. 22.9%; *p* = 0.071) did not differ significantly; results per oocyte retrieval were similar (live birth 29.5% vs. 11.1%; *p* = 0.038). In Firth regression, the crude OR for live birth with flexible vs. fixed initiation was 0.32 (95% CI 0.11–0.89); estimates were similar after adjustment for age and previous IVF attempts, whereas the confidence interval crossed 1 once the number of transferred embryos was added to the model (OR 0.36, 95% CI 0.11–1.04). **Conclusions:** In this small retrospective cohort of women with suboptimal ovarian response, live births were more frequent after fixed than after flexible GnRH antagonist initiation. In view of the small number of events, possible confounding by indication and differences in transfer strategy, this finding is hypothesis-generating and should be tested in a randomised trial.

## 1. Introduction

About 15% of people of reproductive age are affected by infertility, and over 1.5 million IVF and ICSI cycles are carried out worldwide each year [1,2]. Controlled ovarian stimulation (COS) is central to IVF/ICSI, and the success of treatment depends above all on female age and on how many oocytes are obtained [3,4].

Reproductive potential declines with age because of progressive depletion of the ovarian follicular pool and an increased rate of oocyte aneuploidy [5,6]. Moreover, the more oocytes are retrieved, the higher the chance of live birth, both after the fresh transfer and cumulatively per started or aspirated cycle [4].

Adequate ovarian response to COS is therefore a key determinant of treatment outcome. Ovarian response reflects the sensitivity of the follicular cohort to exogenous gonadotropin stimulation during controlled ovarian stimulation [7]. According to the number of oocytes retrieved, women are commonly described as poor (fewer than 4), suboptimal (4 to 9), normal (10 to 15) or high (more than 15) responders [8]. Compared with a normal response, retrieval of only 4 to 9 oocytes carries a significantly lower chance of live birth in all age groups [9,10]. Suboptimal responders are a clinically relevant subgroup because many of these women have apparently normal ovarian reserve markers but still show an inadequate follicular response during stimulation [8,11]. The exact etiology remains unclear, although genetic factors and stimulation-related variables have been proposed [12,13].

Unlike poor responders, suboptimal responders usually have an adequate follicular pool but a reduced follicular response to stimulation. They may therefore be particularly sensitive to differences in luteinizing hormone (LH) exposure, and the timing of GnRH antagonist initiation may matter more in this group than in other patients [14,15,16,17].

During COS, multifollicular development is required to maximize oocyte yield. Pituitary suppression plays a central role in preventing premature LH surge and supporting appropriate follicular development [18].

Gonadotropin-releasing hormone (GnRH) antagonist protocols are widely used in clinical practice because of their favorable safety profile, lower risk of ovarian hyperstimulation syndrome, and shorter treatment duration compared with GnRH agonist protocols [19]. Two main GnRH antagonist initiation strategies are used in routine practice: fixed and flexible protocols. In the fixed protocol, antagonist administration is started on a predefined stimulation day, in the literature typically day 5 or 6. In the flexible protocol, initiation is individualized according to follicular development, usually once the lead follicle has grown to 12–14 mm [20]. The thresholds applied in our unit are described in Methods.

Although fixed and flexible GnRH antagonist protocols have been extensively compared in unselected IVF populations, few studies have specifically evaluated whether protocol timing influences reproductive outcomes in women with suboptimal ovarian response [21,22,23].

Given the biological characteristics of this subgroup, the timing of GnRH antagonist initiation may have a greater impact in this subgroup than in the broader IVF population [12,14]. We therefore compared reproductive outcomes of fixed and flexible GnRH antagonist initiation in women with a suboptimal ovarian response treated with IVF/ICSI.

## 2. Materials and Methods

### 2.1. Study Design and Setting

We conducted a retrospective cohort study at the IVF unit of the Maternity Hospital Petrova (Zagreb, Croatia), covering treatment cycles from January 2019 to February 2020. The Ethical Committee of the Clinical Hospital Center Zagreb approved the study (Class 8.1-24/196-2, No. 02/013 AG); approval, consent and data protection are described in the Institutional Review Board Statement. The study population overlaps with that of our previous study of women in POSEIDON groups 1 and 2, in which the two protocols were compared separately within each POSEIDON group using univariable analyses [24]. The present analysis is restricted to women with a suboptimal response (4–9 oocytes), excluding those with fewer than 4 oocytes, and examines the association between the protocol and live birth with penalised regression adjusted for potential confounders, both per fresh transfer and per oocyte retrieval.

### 2.2. Participants

We screened the records of 419 women who underwent IVF/ICSI with a GnRH antagonist protocol during this period and considered only the first eligible cycle of each woman. Ninety-seven women were excluded because their clinical data were incomplete or because the cycle was performed for fertility preservation. From the remaining 322 women, we selected women with normal ovarian reserve, defined as an antral follicle count (AFC) ≥ 5 and anti-Müllerian hormone (AMH) ≥ 1.2 ng/mL (8.6 pmol/L), in whom 4–9 oocytes were retrieved (suboptimal response; n = 98). Women with low ovarian reserve (AFC < 5 and/or AMH < 1.2 ng/mL; n = 62) and women with fewer than 4 (n = 19), 10–15 (n = 95) or more than 15 oocytes (n = 48) were not included. Both markers were recorded in the hospital information system for every included woman. AMH values are given in pmol/L. Nine of the 98 women did not have a fresh embryo transfer. In the fixed group, fertilisation failed in two women, and one had all embryos frozen because of an elevated progesterone level on the trigger day. In the flexible group, fertilisation failed in two women whose oocytes were all immature, no semen sample was obtained in one, and three had all embryos frozen because of free pelvic fluid around the uterus, which was attributed to the stimulation. The primary analysis therefore included the 89 women who had a fresh transfer (fixed protocol, n = 41; flexible protocol, n = 48), and all 98 women were included in the per-retrieval analysis.

### 2.3. Ovarian Stimulation and Laboratory Procedures

Stimulation began on cycle day 2 with recombinant FSH (rFSH; Gonal-f, Merck Serono), highly purified hMG (Menopur, Ferring) or rFSH combined with recombinant LH in a 2:1 ratio (Pergoveris, Merck Serono). The daily dose (150–300 IU) was chosen individually, taking into account age, ovarian reserve and any earlier response to stimulation. Premature ovulation was prevented with a daily injection of cetrorelix 0.25 mg (Cetrotide, Merck), which was introduced either on stimulation day 6, corresponding to cycle day 7 (fixed protocol), or once the leading follicle measured 13–14 mm (flexible protocol). Approximately eight physicians treated patients during the study period. Each chose the protocol according to individual clinical judgement, without predefined criteria, and to our knowledge all of them used both protocols. Once at least three follicles had reached 18 mm or more, 250 µg of recombinant hCG (Ovitrelle, Merck Serono) was administered, and oocytes were collected transvaginally 36 h later. Insemination was performed by conventional IVF or ICSI, according to semen quality. Embryos were transferred under abdominal ultrasound on day 3 or 4 and at the blastocyst stage (day 5–6). The stage and the number of embryos (one or two) were decided for each woman on the basis of embryo development and quality, independently of the stimulation protocol. Surplus good-quality embryos were vitrified. All women received vaginal micronised progesterone (Utrogestan, Besins Healthcare; 600 mg daily) from the day of oocyte retrieval until week 12 of pregnancy.

### 2.4. Outcomes

The primary outcome was live birth, i.e., delivery of a live-born infant at 24 weeks of gestation or later, expressed per fresh transfer (main analysis) and per oocyte retrieval. Serum β-hCG was tested 14 days after transfer. A positive test without an intrauterine gestational sac was classified as a biochemical pregnancy, an intrauterine sac with fetal cardiac activity at 6–7 weeks as a clinical pregnancy, and the loss of a clinical pregnancy before 22 weeks of gestation as a missed abortion. Secondary outcomes were the numbers of oocytes and metaphase II (MII) oocytes, the transfer strategy, cryopreservation, the total gonadotropin dose, and the duration of stimulation and of antagonist treatment.

### 2.5. Statistical Analysis

**Figure 1:**
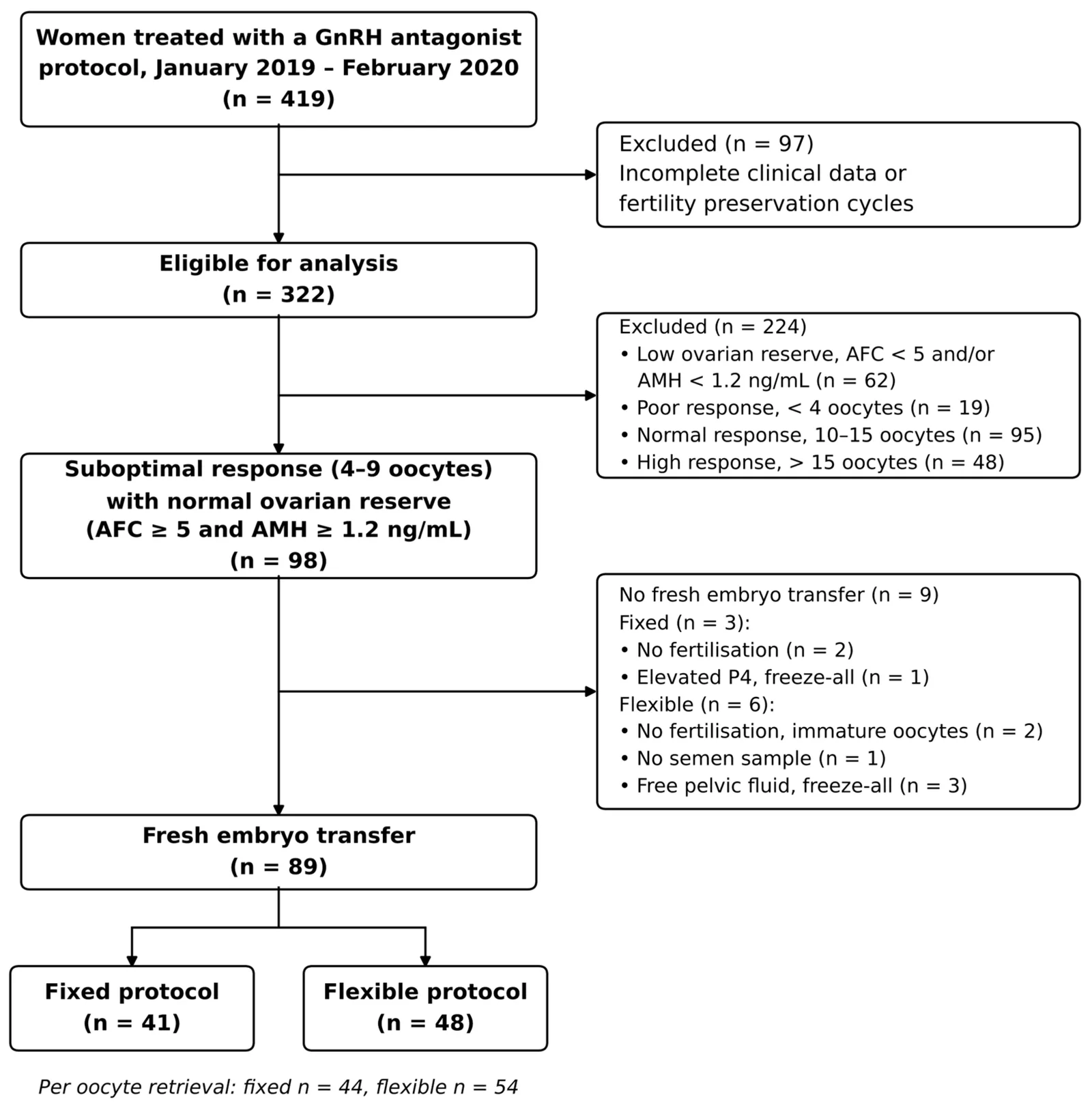
Flow diagram of patient selection. AFC, antral follicle count; AMH, anti-Müllerian hormone; GnRH, gonadotropin-releasing hormone; P4, progesterone.

Continuous data are summarised as median (interquartile range) and were compared with the Mann–Whitney U test. Categorical data are given as n (%) and were compared with Fisher’s exact test or, for the four categories of gonadotropin preparation, with the Fisher–Freeman–Halton test. We report exact *p*-values and regarded a two-sided *p* < 0.05 as significant. Because there were only 19 live births, we used Firth’s penalised logistic regression, which limits small-sample bias, and restricted each model to no more than two covariates in addition to protocol (three in one sensitivity model). The main model was adjusted for female age and the number of previous IVF attempts, both of which are known before treatment. In sensitivity analyses, we adjusted instead for the number of embryos transferred, for blastocyst transfer (yes/no), for both, or for the duration of infertility. Odds ratios (ORs) are presented with 95% profile penalised-likelihood confidence intervals. Missing values were not imputed. An exploratory analysis was stratified by age (<35 vs. ≥35 years). As all eligible women treated during the study period were included, no sample size was calculated in advance. Descriptive statistics and group comparisons were computed with SPSS version 22.0 (IBM Corp., Armonk, NY, USA) and the Firth models with Python 3.13 (NumPy, SciPy). Generative artificial intelligence (Claude, Anthropic) was used to assist with the statistical re-analysis, the preparation of Figure 1 and language editing; all analyses and outputs were checked by the authors.

## 3. Results

Figure 1 shows how the study population was selected. Of the 419 women screened, 322 were eligible, and 98 of the 260 women with normal ovarian reserve (37.7%) had a suboptimal response. After exclusion of the nine women without a fresh transfer (Section 2.2), 89 women remained: 41 treated with the fixed and 48 with the flexible protocol. Among women with normal ovarian reserve, the two protocols were similarly distributed across response categories (poor response 6 vs. 13, suboptimal response 44 vs. 54 and normal response 40 vs. 55 women with the fixed and flexible protocol, respectively; Fisher–Freeman–Halton test, *p* = 0.56). The proportion of women with a suboptimal rather than a normal response was 44/84 (52.4%) with the fixed and 54/109 (49.5%) with the flexible protocol (OR 0.89, 95% CI 0.51–1.58; *p* = 0.77), and the proportion of poor responders among women with fewer than 10 oocytes was 6/50 (12.0%) and 13/67 (19.4%), respectively (OR 1.77, 95% CI 0.62–5.03; *p* = 0.32). The distribution of protocols among high responders could not be assessed.

### 3.1. Baseline Characteristics

Age, AMH, basal FSH and LH, basal progesterone and estradiol, AFC, duration of infertility and number of previous IVF attempts were comparable between groups (Table 1). Baseline and stimulation characteristics were similar when all 98 women, including those without fresh transfer, were analysed (Supplementary Table S1).

### 3.2. Ovarian Stimulation Characteristics

Total gonadotropin dose, duration of stimulation and gonadotropin preparation did not differ between groups. Antagonist administration was started later in the flexible group (median day 8 vs. 7; *p* = 0.003), and antagonist treatment was shorter (median 4 vs. 5 days; *p* < 0.001). On the trigger day, serum progesterone was lower in the flexible group (median 1.60 vs. 2.45 nmol/L; *p* = 0.026), whereas estradiol and endometrial thickness did not differ (Table 2). Trigger-day progesterone and estradiol, which are measured routinely, were available in the study database for 33 and 36 women, respectively.

### 3.3. Embryo Transfer

The numbers of retrieved and MII oocytes did not differ between groups (Table 2). The transfer strategy differed: single-blastocyst transfer was more frequent in the flexible group (41.7% vs. 9.8%; *p* < 0.001), transfer of day-3 embryo(s) or a morula was more frequent in the fixed group (53.7% vs. 25.0%; *p* = 0.008), and two embryos were transferred more often in the fixed group (78.0% vs. 47.9%; *p* = 0.004), with a median of 2 vs. 1 embryo per transfer. Surplus blastocysts were vitrified in 7/41 (17.1%) and 4/48 (8.3%) women (13 vs. 9 blastocysts; *p* = 0.33) (Table 3).

### 3.4. Reproductive Outcomes

Live birth was more frequent in the fixed group (13/41, 31.7% vs. 6/48, 12.5%; *p* = 0.038); positive β-hCG (18/41, 43.9% vs. 13/48, 27.1%; *p* = 0.12) and clinical pregnancy (17/41, 41.5% vs. 11/48, 22.9%; *p* = 0.071) showed the same direction but did not differ significantly. Results per oocyte retrieval were similar (live birth 13/44, 29.5% vs. 6/54, 11.1%; *p* = 0.038). With one fewer live birth in the fixed group, the difference would no longer be statistically significant (fragility index 1). Missed abortion occurred in 4/17 (23.5%) vs. 5/11 (45.5%) clinical pregnancies (*p* = 0.41), a difference based on very small numbers. Live births per embryo transferred were 13/73 (17.8%) vs. 6/71 (8.5%) (*p* = 0.14) (Table 3).

### 3.5. Multivariable Analysis

In Firth regression, the crude OR for live birth with flexible vs. fixed initiation was 0.32 (95% CI 0.11–0.89). After adjustment for age and previous IVF attempts, the OR was 0.32 (95% CI 0.11–0.91; *p* = 0.032), and neither covariate was significantly associated with live birth. Adjustment for the number of embryos transferred gave a similar but less precise estimate (OR 0.36, 95% CI 0.11–1.04; *p* = 0.059), and adjustment for duration of infertility gave an OR of 0.29 (95% CI 0.09–0.86) (Table 4). In an exploratory analysis by age, live birth rates with fixed vs. flexible initiation were 9/16 (56.3%) vs. 2/19 (10.5%) in women < 35 years and 4/25 (16.0%) vs. 4/29 (13.8%) in women ≥ 35 years. After blastocyst transfer, live birth occurred in 7/19 (36.8%) women in the fixed and 4/36 (11.1%) in the flexible group (*p* = 0.035); after transfer of day-3 embryo(s) or a morula, it occurred in 6/22 (27.3%) and 2/12 (16.7%) (*p* = 0.68). Adjustment for blastocyst transfer did not change the OR for flexible initiation (0.31, 95% CI 0.10–0.91), and blastocyst transfer itself was not significantly associated with live birth (OR 1.14, 95% CI 0.39–3.45) (Table 4).

## 4. Discussion

In this retrospective cohort study, we compared fixed and flexible GnRH antagonist initiation protocols in women with suboptimal ovarian response undergoing IVF/ICSI treatment. The main finding was that fixed antagonist initiation was associated with a higher live birth rate than flexible initiation, despite comparable numbers of retrieved and mature oocytes. The estimate was stable across adjusted models (OR 0.29–0.36), but the confidence intervals were wide, the result was fragile, and residual confounding related to the non-randomised design cannot be excluded.

Although fixed and flexible protocols have been widely compared in unselected IVF populations, evidence in this clinically distinct subgroup remains limited [23].

Our results provide new insight into the potential role of pituitary suppression during COS, where individualized stimulation strategies are increasingly recognized as important in assisted reproductive treatment [25]. Optimizing controlled ovarian stimulation remains one of the major challenges in IVF practice [26]. Even when ovarian reserve markers are similar, women may respond differently to gonadotropin stimulation [7]. Suboptimal responders form a group between poor and normal responders. They usually have normal ovarian reserve markers but retrieve fewer oocytes than expected, which suggests a reduced follicular response to stimulation rather than a reduced follicular reserve [10,12,27,28]. Therefore, optimization of stimulation protocols may have a greater impact in this population than in women with consistently normal ovarian response [14].

In our cohort, 98 of 260 women with normal ovarian reserve (37.7%) had a suboptimal ovarian response, a higher proportion than the approximately 27% reported by Hochberg et al., which may reflect differences in patient selection and stimulation practice between centres [29].

Baseline demographic and clinical characteristics were generally comparable between groups, supporting the overall homogeneity of the study population. Age, AMH, duration of infertility and number of previous IVF attempts were similar between groups. However, more embryos were transferred in the fixed group. Adjustment for the number of embryos transferred changed the OR only slightly (from 0.32 to 0.36), and the number of embryos transferred was not itself associated with live birth, but the adjusted confidence interval included 1. Residual confounding by unmeasured factors cannot be excluded. Because of the protocol design, antagonist administration was initiated earlier in the fixed protocol group, resulting in a longer duration of antagonist exposure. Ovarian response was similar between protocols, with similar numbers of retrieved oocytes and mature metaphase II oocytes. Serum progesterone on the trigger day was lower in the flexible group, whereas estradiol did not differ; trigger-day hormone values were not available for all women. Although single hormone measurements cannot fully characterize endocrine dynamics throughout stimulation, these findings may reflect differences in follicular maturation related to the timing of LH suppression [30,31,32].

Follicular synchrony refers to the coordinated growth of multiple follicles during controlled ovarian stimulation, resulting in a more homogeneous follicular cohort with similar developmental potential. The similar numbers of retrieved and mature oocytes suggest that the observed difference is not explained by quantitative ovarian response. Earlier antagonist initiation has been proposed to promote a more synchronised follicular cohort; however, numbers of preovulatory follicles, oocytes and MII oocytes were similar between groups, and this hypothesis could not be evaluated with our data [33,34].

Delayed antagonist initiation may permit prolonged exposure to endogenous LH and estradiol during the early follicular phase, potentially altering the hormonal environment before pituitary suppression is established [35,36]. As proposed by Kolibianakis et al., early exposure of the genital tract to LH and estradiol before antagonist initiation could contribute to differences between pregnant and nonpregnant patients [37]. These early endocrine events in the follicular phase of antagonist cycles may result in earlier progesterone action on the endometrium, leading to endometrial advancement and impaired embryo-endometrium synchrony during fresh embryo transfer [35,38]. Such alterations could reduce implantation potential even when embryo quality is comparable [39].

Serum progesterone on the trigger day was higher in the fixed than in the flexible group. However, progesterone concentrations in both groups were well below thresholds associated with endometrial advancement, and the group with the higher concentrations had the better outcome. Our data therefore do not support an endocrine explanation via premature progesterone elevation. Whether the timing of antagonist initiation affects outcomes through other mechanisms cannot be assessed with the present data.

Fresh blastocyst and single-embryo transfers were more frequent in the flexible group, whereas more embryos were transferred in the fixed group. After blastocyst transfer, live birth was less frequent in the flexible than in the fixed group, although single-blastocyst transfer was more common in the flexible group. Fewer embryos transferred, differences in embryo quality that we could not assess, or unmeasured prognostic factors may explain this finding better than a direct effect of the protocol, and the small numbers in each stratum make it particularly uncertain. Although median age was similar in both groups, differences in embryo aneuploidy cannot be excluded, because embryos were not tested. Most previous randomized trials and meta-analyses have reported comparable reproductive outcomes between fixed and flexible GnRH antagonist protocols in unselected IVF populations [21,22,23]. However, these studies included heterogeneous patient populations with substantial variation in ovarian reserve and ovarian response.

Whether women with suboptimal ovarian response respond differently to antagonist timing than unselected populations remains to be tested. The exploratory finding that the difference was confined to women younger than 35 years should be interpreted with great caution because of the very small numbers.

This study has several strengths. It specifically investigated a clinically well-defined subgroup of women with suboptimal ovarian response, used live birth as the primary outcome measure, and reported outcomes both per embryo transfer and per oocyte retrieval. However, several limitations should be acknowledged. The retrospective design introduces the possibility of selection bias and residual confounding, while protocol allocation was based on physician preference rather than randomization. Physicians may have chosen the protocol according to patient characteristics that were not recorded, so selection bias and confounding by indication cannot be excluded. The identity of the treating physician and the calendar date of each cycle were not recorded, so confounding by physician or by changes in practice over time could not be assessed. Another important limitation is that the study population was defined by the number of oocytes retrieved, which is itself a consequence of ovarian stimulation. If the timing of antagonist initiation influenced oocyte yield, women with 4–9 oocytes in the fixed and flexible groups might not represent comparable populations, which could introduce selection bias. The distribution of protocols across poor, suboptimal and normal responders did not differ significantly, and the median numbers of retrieved and mature oocytes were identical in both groups; nevertheless, because the distribution among high responders and outcomes in the whole cohort could not be analysed, this source of bias cannot be excluded. In addition, fertilisation, blastocyst formation and embryo quality were not included in the study database and could not be analysed. Only 19 live births occurred, so the multivariable analysis is exploratory, the confidence intervals are wide and the primary result is fragile (fragility index 1); no a priori sample-size calculation was performed. More embryos were transferred in the fixed group, which may have contributed to the difference despite adjustment. Outcomes of subsequent frozen embryo transfers were not available, so cumulative live birth rate could not be assessed. Therefore, these findings should be interpreted as hypothesis-generating and require confirmation in adequately powered prospective randomized studies.

## 5. Conclusions

In this small retrospective cohort of women with suboptimal ovarian response, live births were more frequent after fixed than after flexible GnRH antagonist initiation. In view of the small number of events, possible confounding by indication and differences in transfer strategy, this finding is hypothesis-generating and should be tested in a randomised trial.

## Figures and Tables

**Table 1:** Baseline characteristics of women with suboptimal ovarian response treated with fixed or flexible GnRH antagonist initiation.

Variable	Fixed (n = 41)	Flexible (n = 48)	*p*
Age, years	36.0 (33.5–40.0)	36.0 (33.2–39.0)	0.99
Duration of infertility, years	3 (2–4)	3 (2–4)	0.72
Previous IVF attempts, n	1 (0–1)	1 (0–2)	0.27
FSH, IU/L	7.0 (5.8–8.5)	6.8 (5.0–8.5)	0.40
LH, IU/L	4.7 (3.8–6.2)	4.4 (3.2–6.1)	0.47
AMH, pmol/L	13.0 (9.6–20.5)	14.0 (9.4–19.5)	0.77
AFC, n	9.5 (6.5–13.8)	9 (6–11)	0.77
Basal P4, nmol/L	0.80 (0.60–1.20)	1.05 (0.90–1.47)	0.088
Basal E2, pg/mL	183 (116–234)	161 (111–195)	0.45

Values are median (interquartile range); groups were compared with the Mann–Whitney U test. AFC, antral follicle count; AMH, anti-Müllerian hormone; E2, estradiol; FSH, follicle-stimulating hormone; IQR, interquartile range; LH, luteinizing hormone; P4, progesterone. Previous IVF attempts: 0 = first IVF/ICSI cycle.

**Table 2:** Ovarian stimulation and laboratory characteristics.

Variable	Fixed (n = 41)	Flexible (n = 48)	*p*
Total gonadotropin dose, IU	2250 (1969–2981)	2400 (2006–2700)	0.88
Duration of stimulation, days	10 (9–11)	10 (9–11)	0.053
Day of antagonist initiation (cycle day)	7 (7–7)	8 (7–9)	0.003
Duration of antagonist, days	5 (4–6)	4 (3–5)	<0.001
Preovulatory follicles, n	7 (6–8)	6 (5–8)	0.29
Endometrium on trigger day, mm	10.0 (9.0–11.7)	10 (9–12)	0.46
P4 on trigger day, nmol/L	2.45 (2.12–3.42)	1.60 (1.00–2.70)	0.026
E2 on trigger day, pg/mL	1124 (801–1508)	885 (529–1559)	0.52
Oocytes retrieved, n	6 (4–7)	6 (4–7)	0.84
MII oocytes, n	4.5 (3.0–6.0)	5.0 (3.5–6.0)	0.71
Embryos transferred, n	2 (2–2)	1 (1–2)	0.004
Gonadotropin preparation			0.36
rFSH, n (%)	35 (85.4%)	40 (83.3%)	
hMG, n (%)	5 (12.2%)	5 (10.4%)	
rFSH + hMG, n (%)	1 (2.4%)	0 (0.0%)	
rFSH + rLH, n (%)	0 (0.0%)	3 (6.3%)	

Values are median (interquartile range) or n (%). Continuous variables were compared with the Mann–Whitney U test and gonadotropin preparation with the Fisher–Freeman–Halton exact test. Antagonist initiation is given as cycle day; stimulation started on cycle day 2. E2, estradiol; P4, progesterone; hMG, highly purified human menopausal gonadotropin; rFSH, recombinant FSH; rLH, recombinant LH; MII, metaphase II.

**Table 3:** Embryo transfer strategy and reproductive outcomes.

Variable	Fixed	Flexible	*P*
***Transfer strategy***			
Single blastocyst, n (%)	4 (9.8%)	20 (41.7%)	<0.001
Double blastocyst, n (%)	15 (36.6%)	16 (33.3%)	0.82
Day-3 embryo(s) or morula, n (%)	22 (53.7%)	12 (25.0%)	0.008
Two embryos transferred, n (%)	32 (78.0%)	23 (47.9%)	0.004
Embryos transferred, total/live births per embryo	73/13 (17.8%)	71/6 (8.5%)	0.14
***Cryopreservation***			
Surplus blastocysts vitrified, n (%)	7 (17.1%)	4 (8.3%)	0.33
Vitrified blastocysts, total	13	9	
***Outcomes per embryo transfer (fixed n = 41, flexible n = 48)***			
Positive β-hCG, n (%)	18 (43.9%)	13 (27.1%)	0.12
Biochemical pregnancy, n (%)	1 (2.4%)	2 (4.2%)	1.00
Clinical pregnancy, n (%)	17 (41.5%)	11 (22.9%)	0.071
Missed abortion, n (%)	4 (9.8%)	5 (10.4%)	1.00
Live birth, n (%)	13 (31.7%)	6 (12.5%)	0.038
Missed abortion per clinical pregnancy, n (%)	4 (23.5%)	5 (45.5%)	0.41
***Outcomes per oocyte retrieval (fixed n = 44, flexible n = 54)***			
Positive β-hCG, n (%)	18 (40.9%)	13 (24.1%)	0.085
Biochemical pregnancy, n (%)	1 (2.3%)	2 (3.7%)	1.00
Clinical pregnancy, n (%)	17 (38.6%)	11 (20.4%)	0.071
Missed abortion, n (%)	4 (9.1%)	5 (9.3%)	1.00
Live birth, n (%)	13 (29.5%)	6 (11.1%)	0.038

Values are n (%) unless stated otherwise; groups were compared with Fisher’s exact test. All women in the per-transfer analysis had a fresh embryo transfer. Outcomes per oocyte retrieval include the 9 women without transfer (3 fixed, 6 flexible), who by definition had no pregnancy.

**Table 4:** Firth penalised logistic regression for live birth: crude and adjusted odds ratios for flexible versus fixed GnRH antagonist initiation.

Model/Variable	OR (95% CI)	*P*
***Crude***		
Flexible vs. fixed protocol	0.32 (0.11–0.89)	0.029
***Adjusted for previous IVF attempts***		
Flexible vs. fixed protocol	0.34 (0.11–0.93)	0.036
Previous IVF attempts (per attempt)	0.97 (0.55–1.45)	0.89
***Adjusted for previous IVF attempts and age***		
Flexible vs. fixed protocol	0.32 (0.11–0.91)	0.032
Previous IVF attempts (per attempt)	1.08 (0.61–1.65)	0.76
Age (per year)	0.91 (0.80–1.02)	0.099
***Adjusted for number of embryos transferred***		
Flexible vs. fixed protocol	0.36 (0.11–1.04)	0.059
Embryos transferred (per embryo)	1.37 (0.44–4.59)	0.59
***Adjusted for duration of infertility***		
Flexible vs. fixed protocol	0.29 (0.09–0.86)	0.025
Duration of infertility (per year)	0.79 (0.48–1.09)	0.17
***Adjusted for age***		
Flexible vs. fixed protocol	0.32 (0.11–0.90)	0.031
Age (per year)	0.91 (0.81–1.02)	0.094
***Adjusted for blastocyst transfer***		
Flexible vs. fixed protocol	0.31 (0.10–0.91)	0.032
Blastocyst transfer (yes vs. no)	1.14 (0.39–3.45)	0.82
***Adjusted for blastocyst transfer and number of embryos transferred***		
Flexible vs. fixed protocol	0.35 (0.11–1.04)	0.059
Blastocyst transfer (yes vs. no)	1.15 (0.40–3.46)	0.80
Embryos transferred (per embryo)	1.37 (0.45–4.60)	0.58

Firth’s penalised logistic regression; dependent variable: live birth (yes/no). Blastocyst transfer: yes = at least one blastocyst transferred; no = day-3 embryo(s) or morula. OR, odds ratio; CI, profile penalised-likelihood confidence interval. Because only 19 live births occurred, models include at most two covariates in addition to protocol (three in the last sensitivity model); the analysis is exploratory. Continuous covariates were centred. Each model included all women with data for the covariates in that model.

## Data Availability

The data presented in this study are available upon reasonable request from the corresponding author.

## Supplementary Materials

The following supporting information can be downloaded at https://www.mdpi.com/article/10.3390/jcm15218497/s1. Supplementary Table S1. Baseline and ovarian stimulation characteristics of all women with suboptimal ovarian response (per oocyte retrieval, n = 98), including the 9 women without fresh embryo transfer.
