# Supplementary material for: Fixed Versus Flexible GnRH Antagonist Initiation in Women with Suboptimal Ovarian Response Undergoing IVF/ICSI: A Retrospective Cohort Study
Source: J Clin Med. 2026 Nov 1;15(21):8497. doi: 10.3390/jcm15218497 (PMC13764640; doi:10.3390/jcm15218497)
Supplement: Supplementary file 1 [file jcm-15-08497-s001.zip › jcm-4553805-supplementary.pdf]

Supplementary Table S1. Baseline and ovarian stimulation characteristics of all women with suboptimal ovarian response (per oocyte retrieval, n = 98), including the 9 women without fresh embryo transfer.

Variable	Fixed (n = 44)	Flexible (n = 54)	p
Baseline characteristics			
Age, years	36.0 (33.0–39.8)	36.0 (33.8–39.2)	0.63
Duration of infertility, years	3 (2–4)	3 (2–4)	0.68
Previous IVF attempts, n	0 (0–1)	1 (0–2)	0.38
FSH, IU/L	6.9 (6.0–8.0)	6.8 (5.0–8.5)	0.35
LH, IU/L	4.3 (3.6–6.1)	4.8 (3.3–6.1)	0.96
AMH, pmol/L	13.0 (9.6–20.0)	14.0 (9.0–20.4)	0.82
AFC, n	10 (8–12)	9 (7–11)	0.73
Basal P4, nmol/L	0.90 (0.65–1.15)	1.00 (0.90–1.35)	0.11
Basal E2, pg/mL	183 (110–255)	150 (104–188)	0.21
Ovarian stimulation			
Total gonadotropin dose, IU	2250 (1950–2925)	2400 (2025–2700)	0.98
Duration of stimulation, days	10 (9–11)	10 (9–11)	0.083
Day of antagonist initiation (cycle day)	7 (7–7)	8 (7–9)	<0.001
Duration of antagonist, days	5 (4–6)	4 (3–5)	<0.001
Preovulatory follicles, n	7 (6–8)	6 (5–9)	0.51
Endometrium on trigger day, mm	10.0 (9.0–11.8)	10.0 (8.9–12.0)	0.44
P4 on trigger day, nmol/L	2.45 (2.12–3.42)	1.60 (1.02–2.68)	0.023
E2 on trigger day, pg/mL	1135 (802–1522)	1061 (529–1755)	0.97
Oocytes retrieved, n	6 (4–7)	6 (5–7)	0.50
MII oocytes, n	4 (3–6)	5 (4–6)	0.27
Gonadotropin preparation			0.54
rFSH, n (%)	38 (86.4%)	45 (83.3%)	
hMG, n (%)	5 (11.4%)	5 (9.3%)	
rFSH+hMG, n (%)	1 (2.3%)	1 (1.9%)	
rFSH+rLH, n (%)	0 (0.0%)	3 (5.6%)	

Values are median (interquartile range) or n (%). Continuous variables were compared with the Mann–Whitney U test and gonadotropin preparation with the Fisher–Freeman–Halton exact test. Antagonist initiation is given as cycle day. AFC, antral follicle count; AMH, anti-Müllerian hormone; E2, estradiol; FSH, follicle-stimulating hormone; hMG, highly purified human menopausal gonadotropin; LH, luteinizing hormone; MII, metaphase II; P4, progesterone; rFSH, recombinant FSH; rLH, recombinant LH. Previous IVF attempts: 0 = first IVF/ICSI cycle.
